# The use of singleplex and nested PCR to detect *Batrachochytrium dendrobatidis* in free-living frogs

**DOI:** 10.1590/S1517-838246246220140110

**Published:** 2015-06-01

**Authors:** Selene Dall'Acqua Coutinho, Julieta Catarina Burke, Catia Dejuste de Paula, Miguel Trefaut Rodrigues, José Luiz Catão-Dias

**Affiliations:** 1 Universidade Paulista, Laboratório de Biologia Molecular e Celular, Universidade Paulista, São Paulo, SP, Brasil, Laboratório de Biologia Molecular e Celular, Universidade Paulista, São Paulo, SP, Brazil.; 2 Universidade de São Paulo, Laboratório de Patologia Comparada de Animais Selvagens, Faculdade de Medicina Veterinária e Zootecnia, Universidade de São Paulo, São Paulo, SP, Brasil, Laboratório de Patologia Comparada de Animais Selvagens, Faculdade de Medicina Veterinária e Zootecnia, Universidade de São Paulo, São Paulo, SP, Brazil.; 3 Universidade de São Paulo, Instituto de Biociências, Universidade de São Paulo, São Paulo, SP, Brasil, Instituto de Biociências, Universidade de São Paulo, São Paulo, SP, Brazil.

**Keywords:** chytridiomycosis, *Batrachochytrium dendrobatidis*, frogs, Brazilian Atlantic Forest, PCR

## Abstract

Many microorganisms are able to cause diseases in amphibians, and in the past few years one of the most reported has been *Batrachochytrium dendrobatidis.* This fungus was first reported in Brazil in 2005; following this, other reports were made in specimens deposited in museum collections, captive and free-living frogs. The aim of this study was to compare singleplex and nested-PCR techniques to detect *B. dendrobatidis* in free-living and apparently healthy adult frogs from the Brazilian Atlantic Forest. The sample collection area was a protected government park, with no general entrance permitted and no management of the animals there. Swabs were taken from the skin of 107 animals without macroscopic lesions and they were maintained in ethanol p.a. Fungal DNA was extracted and identification of *B. dendrobatidis* was performed using singleplex and nested-PCR techniques, employing specific primers sequences. *B. dendrobatidis* was detected in 61/107 (57%) and 18/107 (17%) animals, respectively by nested and singleplex-PCR. Nested-PCR was statistically more sensible than the conventional for the detection of *B. dendrobatidis* (Chi-square = 37.1; α = 1%) and the agreement between both techniques was considered just fair (Kappa = 0.27). The high prevalence obtained confirms that these fungi occur in free-living frogs from the Brazilian Atlantic Forest with no macroscopic lesions, characterizing the state of asymptomatic carrier. We concluded that the nested-PCR technique, due to its ease of execution and reproducibility, can be recommended as one of the alternatives in epidemiological surveys to detect *B. dendrobatidis* in healthy free-living frog populations.

## Introduction

Of the 6,200 species of living anurans (Frost, 2013), approximately 30% are endangered, presenting the greatest risk situation on the planet (IUCN-ASG, 2013). The decline and extinction of amphibians that have been detected over the last few decades has no precedent in the last millennia (Stuart *et al.*, 2004).

Many microorganisms cause diseases in amphibians, and in the past few years, one of the most widely reported has been *Batrachochytrium dendrobatidis*, a Chytridiomycete fungus of the order Rhizophydiales (Longcore *et al.*, 1999; de Hoog *et al.*, 2004). Chytridiomycosis is a highly contagious disease that occurs worldwide and can lead to a fatal evolution (Fisher *et al.*, 2009; James *et al.*, 2009; Voyles *et al.*, 2009), and chytridiomycosis has been added to the list of compulsory notifiable diseases by the World Organization for Animal Health (OIE, 2013).

These fungi degrade cellulose and keratin, and the infectious form of *B. dendrobatidis* is the zoospore (Longcore *et al.*, 1999; de Hoog *et al.*, 2004).

Although there is no consensus on the infective dose for amphibians, the presence of only a single zoospore may be sufficient for the installation and multiplication of the fungus, and infections with a small inoculum (100 zoospores) can cause death in certain species of frogs (Berger *et al.*, 1999; Daszak *et al.*, 1999; James *et al.*, 2009).

These fungi grow within and are able to damage keratinized cells. In histological sections, skin affected by the fungus shows a thickening of the stratum corneum and the presence of sporangia; the evolution of infection causes hyperkeratosis and alterations in the normal epidermis architecture (Longcore *et al.*, 1999; Hyatt *et al.*, 2007).

Due to the role of the skin in these animals, previous studies have suggested that infection with *B. dendrobatidis* may affect cutaneous osmoregulation because the changes introduced by the fungus inhibit sodium absorption, compromising the conduction of water and electrolytes and the functions of osmoregulation (Voyles *et al.*, 2009).

This fungus was first described in 1999 (Longcore *et al.*, 1999), and since then, conclusive evidence has suggested that *B. dendrobatidis* infection could be related to the decline of amphibian populations throughout the world (Daszak *et al.*, 1999; James *et al.*, 2009; Voyles *et al.*, 2009). Factors related to human impacts, such as climate change, pollution, deforestation and expansion of crop lands, can together contribute to the transmission of the disease and occurrence of outbreaks (Daszak *et al.*, 1999; Garner *et al.*, 2006; Fisher *et al.*, 2009; James *et al.*, 2009).

The first report of chytridiomycosis in Brazil occurred in tadpoles of the Brazilian Atlantic Forest, which presented oral deformities (Carnaval *et al.*, 2005; Toledo *et al.*, 2006a). Later, chytridiomycosis was observed in specimens deposited in museum collections (Carnaval *et al.*, 2006; Toledo *et al.*, 2006b) and captive (de Paula *et al.*, 2010) and free-living amphibians (Ramalho *et al.*, 2013). *B. dendrobatidis* infection has also been reported in Brazilian farm bullfrogs (Schloegel *et al.*, 2009) and species of frogs inhabiting different altitudes (Grundler *et al.*, 2012). However, there is no record of natural outbreaks in free-living anurans in Brazil (OIE, 2013).

Because Brazil hosts the highest diversity of amphibians in the world, with 946 species (Segalla *et al.*, 2012), and the existence of the fungus in Brazil has been verified, epidemiological surveys in the wild are urgently needed to provide more information about the presence of *B. dendrobatidis* in different species and ecosystems (Grundler *et al.*, 2012; Ramalho *et al.*, 2013).

The most commonly used diagnostic tests for identifying *B. dendrobatidis* utilize histological methods and molecular biology, particularly singleplex, nested or real-time PCR (Berger *et al.*, 1999; Annis *et al.*, 2004; Retallick *et al.*, 2006; Hyatt *et al.*, 2007; Kirshtein *et al.*, 2007). Molecular diagnostic techniques use specific primers to detect fungus, and recent publications have considered real-time and nested PCR more sensitive than singleplex (Boyle *et al.*, 2004; Garner *et al.*, 2006; Goldberg *et al.*, 2007; Garland *et al.*, 2011).

Therefore, this study aimed to compare the performance of singleplex and nested PCR in detecting *B. dendrobatidis* in free-living and apparently healthy adult frogs from the Brazilian Atlantic Forest along the São Paulo state coast.

## Materials and Methods

Samples were collected at the Boracéia Biological Station (23°39′14.10″ S, 45°53′22.53″ W), a protected area maintained in nearly pristine condition by the Museum of Zoology, University of São Paulo, São Paulo state, with no general entrance permitted for visitors. No animal management occurs at this site. Anurans were captured using non-powdered latex gloves and transported in individual plastic bags containing air to the laboratory, where they were physically restrained for sampling. Swabs were taken from the skin of 107 free-living adult frogs (13 genera and 28 species) showing no macroscopic lesions. Sterile swabs were rubbed over the entire body of the animals, preserved in ethanol p.a. and maintained refrigerated (Daszak *et al.*, 1999). All necessary ethical and environmental permits and principles were observed.

The extraction of fungal rDNA was performed with the *PureLink* ^TM^ *Genomic DNA Mini Kit* (Invitrogen^TM^, Carlsbad, CA, USA) according to the manufacturer's guidelines. The identification of *B. dendrobatidis* was performed by singleplex (conventional) PCR, with a limit detection of approximately 10 fungus zoospores, using primers that amplify a specific sequence of rDNA of *B. dendrobatidis*: Bd1a (5′CAGTGTGCCATATGTCACG3′) and Bd2a (5′CATGGTTCATATCTGTCCAG3′) (Annis *et al.*, 1999). The reactions were performed in a volume of 25 μL with 5 μL of DNA (50 ng), 2.5 μL of each primer (1 μM), 12.5 μL (1X) of *Go Taq® Hot Start Green Master Mix* (Promega, Madison, WI, USA) and 2.5 μL of nuclease-free water according to the manufacturer's guidelines.

The amplification reactions were performed in an *Eppendorf Mastercycler Gradient*® 5333 thermocycler (Eppendorf, Hamburg, Germany) and consisted of an initial denaturation at 93°C for 10 min, followed by 30 cycles of 45 s at 93 °C, 45 s at 60 °C, 1 min at 72 °C and a final extension for 10 min at 72 °C. After amplification, the samples were submitted to electrophoresis on agarose gel (1%), stained with ethidium bromide (0.5 μg/mL), visualized on a UV transilluminator and photographed using the *Gel Logic 200 Kodak* system (Eastman Kodak Co., Rochester, NY, USA). Nested PCR was performed by repeating all of the procedures described above in the products obtained by singleplex PCR. Positive *B. dendrobatidis* DNA obtained from the *Amphibian Disease Laboratory* of *San Diego Zoo,* California, USA, was used (Dr. Allan Pessier)*.* The results of the two techniques employed were compared using Chi-square (α = 1%) and concordance analysis (κ - Kappa) tests (Siegel and Castellan, 1988).

## Results

**Figure 1:**
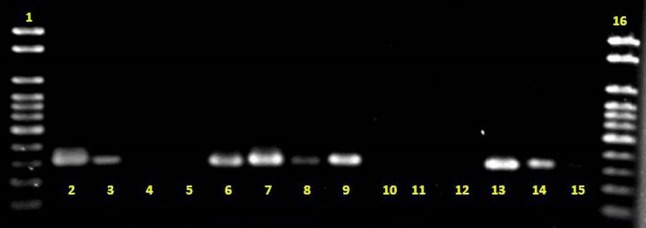
Electrophoresis on agarose gel. 1 and 16: ladder (100-bp); 2: positive control for *B. dendrobatidis* (300-bp); 3, 6, 7, 8, 9, 13 and 14: positive samples for *B. dendrobatidis* (300-bp); 4, 5, 10, 11, 12 and 15: negative samples for *B. dendrobatidis*.

We detected *B. dendrobatidis* in 28 different species of frogs. *B. dendrobatidis* was detected in 61/107 (57%) and 18/107 (17%) animals, respectively, by nested and singleplex PCR (Table 1 and Fig. 1). Nested PCR was more sensitive than singleplex PCR for detecting *B. dendrobatidis* in healthy frogs (Chi-square = 37.1; α = 1%) and the agreement between both techniques was considered only fair (Kappa = 0.27).

**Table 1:** Detection of *Batrachochytrium dendrobatidis* in healthy free-living frogs from the Brazilian Atlantic Forest by singleplex and nested PCR.

Species	Number	Singleplex PCR	Nested PCR
*Hylodes asper*	6	Negative	Positive
*Leptodactylus latrans*	3	Negative	Positive
*Scinax alter*	2	Positive	Positive
*Adenomera marmorata*	1	Negative	Positive
*Dendropsophus minutus*	1	Positive	Positive
*Ischnocnema randorum*	1	Positive	Positive
*Ischnocnema parva*	1	Positive	Positive
*Aplastodiscus arildae*	1	Negative	Positive
*Hypsiboas faber*	3	Positive	Positive
*Aplastodiscus leucopygius*	1	Positive	Positive
*Scinax crospedosfilus*	3	Negative	Positive
*Bokermannohyla astartea*	2	Negative	Positive
*Hylodes asper*	2	Positive	Positive
*Hypsiboas bischoffi*	1	Negative	Positive
*Aplastodiscus albosignatus*	1	Negative	Positive
*Dendropsophus minutus*	1	Negative	Positive
*Hypsiboas bischoffi*	2	Positive	Positive
*Hypsiboas polytaenius*	2	Positive	Positive
*Hypsiboas polytaenius*	4	Negative	Positive
*Physalaemus cuvieri*	1	Negative	Positive
*Phyllomedusa burmeisteri*	3	Negative	Positive
*Phyllomedusa rohdei*	1	Negative	Positive
*Trachycephalus mesophaeus*	2	Negative	Positive
*Scinax fuscovarius*	1	Negative	Positive
*Hypsiboas semilineatus*	2	Negative	Positive
*Trachycephalus mesophaeus*	1	Positive	Positive
*Ischnocnema parva*	1	Negative	Positive
*Scinax brieni*	1	Negative	Positive
*Hylodes phyllodes*	1	Positive	Positive
*Ischnocnema guentheri*	1	Negative	Positive
*Bokermannhoyla hylax*	1	Negative	Positive
*Bokermannohyla circundata*	1	Positive	Positive
*Scinax hayii*	3	Negative	Positive
*Rhinella ornata*	3	Negative	Positive
Total		18	61

The remaining 46 sampled animals tested negative for the presence of *B. dendrobatidis*.

## Discussion

The detection of *B. dendrobatidis* in 28 different species of frogs is consistent with previous results that observed the fungus virtually worldwide, with disease occurrence in a broad range of hosts (Hyatt *et al.*, 2007; Fisher *et al.*, 2009; James *et al.*, 2009; Grundler *et al.*, 2012).

Other studies conducted in Brazil have observed the occurrence of the fungus in the Brazilian Atlantic Forest (Carnaval *et al.*, 2006; Toledo *et al.*, 2006b; Schloegel *et al.*, 2009), as well as in animals from the Cerrado (Brazilian savannah) (Ramalho *et al.*, 2013). Our study confirmed the high prevalence of the fungus in Brazil.

The analyzed animals were from the wild and had no macroscopic lesions or clinical signs of the disease; thus, they were characterized as asymptomatic carriers and could be sources of infection for other animals. Declines in amphibian populations in the Brazilian Atlantic Forest have been reported, and the affected sites include the present study area; however, the causes of these decreases have not yet been determined (Verdade *et al.*, 2013). It would be interesting to expand epidemiological surveys on the presence of *B. dendrobatidis* to other Brazilian biomes to obtain more information on the distribution of these fungi in Brazil.

*B. dendrobatidis* may be able to live saprophytically on keratin in nature if other components of the ecosystem limit the growth of bacteria and phycomycetes (Longcore *et al.*, 1999). Amphibians may maintain *B. dendrobatidis* in their skin, and when an imbalance in the relationship between fungi and host occurs, these fungi can act as opportunistic microorganisms, potentially causing outbreaks of chytridiomycosis similar to those observed in other countries (Stuart *et al.*, 2004; James *et al.*, 2009; Voyles *et al.*, 2009).

The positivity of *B. dendrobatidis* observed using nested PCR (57%) was significantly higher than that found using singleplex PCR (17%), suggesting that nested PCR should be the first technique used to detect the fungus between the two tested. These results are in agreement with recent publications that have considered nested and real-time PCR more sensitive than singleplex PCR (Boyle *et al.*, 2004; Garner *et al.*, 2006; Goldberg *et al.*, 2007; Garland *et al.*, 2011).

Nested PCR consists of PCR execution with the product obtained by singleplex PCR, allowing fungus detection even when few in number, and nested PCR does not require special equipment. However, nested PCR does not allow quantification (Garner *et al.*, 2006; Goldberg *et al.*, 2007). Real-time PCR has been recommended due to its high sensitivity and ability to quantify of the number of fungi; however, it requires special equipment (Boyle *et al.*, 2004; Garland *et al.*, 2011).

We conclude that *B. dendrobatidis* is very prevalent in the anurans living in the sampled area and that nested PCR can be used as an alternative to epidemiological surveys to detect these fungi on healthy free-living frog populations.
